# The clinical practice and dosimetric outcome of the manual adaptive planning during definitive radiotherapy for cervical cancer

**DOI:** 10.1007/s00432-024-05809-z

**Published:** 2024-05-27

**Authors:** Yi-Wei Wang, Min Chen, Wen-Tong Shen, Hao-Ping Xu

**Affiliations:** Department of Radiation Oncology, Ruijin Hospital, Shanghai Jiaotong University, School of Medicine, No.197 Rui Jin Er Rd, Shanghai, 200025 China

**Keywords:** Locally advanced cervical cancer, Radiotherapy, Manual adaptive plan, Dosimetry, Clinical predictors

## Abstract

**Propose:**

To evaluate the advantage of the manual adaptive plans comparing to the scheduled plans, and explored clinical factors predicting patients suitable for adaptive strategy.

**Methods and materials:**

Eighty two patients with weekly online cone-beam computed tomography (CBCT) were enrolled. The re-CT simulation was performed after 15 fractions and a manual adaptive plan was developed if a significant deviation of the planning target volume (PTV) was found. To evaluate the dosimetric benefit, D98, homogeneity index (HI) and conformity index (CI) for the planning target volume (PTV), as well as D2cc of the bowel, bladder, sigmoid and rectum were compared between manual adaptive plans and scheduled ones. The clinical factors influencing target motion during radiotherapy were analyzed by chi-square test and logistic regression analysis.

**Results:**

The CI and HI of the manual adaptive plans were significantly superior to the scheduled ones (*P* = 0.0002, 0.003, respectively), demonstrating a better dose coverage of the target volume. Compared to the scheduled plans, D98 of the manual adaptive plans increased by 3.3% (*P* = 0.0002), the average of D2cc to the rectum, bladder decreased 0.358 Gy (*P* = 0.000034) and 0.240 Gy (*P* = 0.03), respectively. In addition, the chi-square test demonstrated that age, primary tumor volume, and parametrial infiltration were the clinical factors influencing target motion during radiotherapy. Multivariate analysis further identified the large tumor volume (≥ 50cm^3^, OR = 3.254, *P* = 0.039) and parametrial infiltration (OR = 3.376, *P* = 0.018) as the independent risk factors.

**Conclusion:**

We found the most significant organ motion happened after 15 fractions during treatment. The manual adaptive plans improved the dose coverage and decreased the OAR doses. Patients with bulky mass or with parametrial infiltration were highly suggested to adaptive strategy during definitive radiotherapy due to the significant organ motion.

**Supplementary Information:**

The online version contains supplementary material available at 10.1007/s00432-024-05809-z.

## Introduction

Locally advanced cervical cancer (LACC), which is defined as stage IB3-IVA according to the 2018 International Federation of Gynecology and Obstetrics (FIGO) staging system, accounts for more than 80% of all cases in patients with cervical cancer (Sung et al. 2021). Concurrent chemoradiotherapy is the standard of care for locally advanced cervical cancer (Cohen et al. 2019; Monk et al. 2007). However the tumor regression and deformation combined with organ motion during the course of fractionated RT remain challenges in clinical practice of IMRT use.

Often a significant reduction of cervix tumor is observed during the 5 weeks of radiotherapy (RT) (Mayr et al. 2002, 1996). Besides the tumor regression, previous studies also demonstrated the intrafraction movements of pelvic organ, especially the movements of uterus, the filling status of bladder and rectum might result in variations in position and shape of the clinical target volume (CTV), and eventually cause geographical miss of the target and unnecessary volumes of organs-at-risk (OARs) included into high dose regions (Beadle et al. 2009; Chan et al. 2008; Lee et al. 2007). The most effective way to date is online adaptive radiotherapy (ART), which uses an imaging feedback loop to correct the deviation caused by tumor shrinkage and organ motion and modify the treatment plan accordingly (Shelley et al. 2021; Hall et al. 2022; Yan et al. 1997).

Despite the real-time online ART has provided improved clinical benefits (Tan et al. 2019), this technique needs intensive clinical effort, facing big challenges with clinical implementation, which make its utilization relatively limited (Qin et al. 2015), especially in low- and middle-income countries where cervical cancer has a greater prevalence. The time spent throughout the online ART workflow has been always an important consideration especially in a busy radiation oncology clinic. Radiation oncologists spend more time (usually more than 30 min per fraction) on verification of the recontouring and approving the replanning at each treatment fraction, while the patient was lying on the LINAC treatment couch during the whole process which would cause further changes of the bladder and rectum filling. In addition, all adaptive plans are automatically created and cannot be adjusted manually to better meet the whole team needs.

It would be beneficial to find an alternative workflow to avoid recontouring for every adaptive plan. A number of studies showed the largest uterus motion happened in the 2nd or 3rd week of treatment (Lee et al. 2007), and the most of tumor regression occurs about 21 days, or after 30.8 Gy of treatment (Lee et al. 2004, and Bunt et al. 2006). Thus, we proposed an alternative offline ART, the manual adaptive radiotherapy, which only required one more CT simulation after 15 fractions during treatment, and the manual adaptive plan was created by conventional treatment planning systems. The offline ART course could be more easily implemented in clinics.

The goal of our study was to verify the appropriate time for manual adaptive planning during the fractionated treatment, compare the dosimetric outcomes of manual adaptive plans to the scheduled plans, and explore the clinical factors to guide patient selection for ART.

## Materials and methods

### Patients and treatment

Consecutive patients with intact cervical cancer undergoing definitive treatment at our department who agreed to undergo twice CT simulation scans during treatment participated in this study. Between August 2019 and November 2022, 82 patients with histologically confirmed cervical cancer were included. Staging was performed according to the 2018 International Federation of Gynecology and Obstetrics (FIGO) classification. The characteristic of patients, including age, BMI, FIGO stage, tumor size, histological type, and courses of concurrent chemotherapy, were recorded.

Patients underwent CT simulation for treatment planning (initial CT) in supine position with custom immobilization and 3-mm slice thickness. All patients were treated on a linear accelerator equipped with kilo-voltage CBCT (kV-CBCT) with IMRT using seven to nine coplanar fields and 6-MV photons. Treatment was planned using the Eclipse Treatment Planning System version 13.0 (Varian Medical Systems, Palo Alto, CA). In accordance with our treatment protocol all the patients received a combination of external beam radiation therapy (EBRT) with concurrent cisplatin of 40 mg/m^2^ weekly, followed by high-dose-rate brachytherapy 6 Gy per fraction for 5 fractions.

The delineation of the clinical target volume and organs-at-risk is in accordance with RTOG consensus guidelines. The dose given to PTV was 45–50.4 Gy (25 or 28 fractions of 1.8 Gy), five fractions a week. Simultaneous integrated boost (SIB) of the parametrium and positive lymph nodes were applied if needed. And all patients received the high-dose-rate brachytherapy 6 Gy per fraction for 5 fractions. The total dose achieved 85–90 Gy (EQD2, biologically equivalent dose of 2 Gy per fraction).

All patients received at least a weekly cone-beam computed tomography (CBCT) scan throughout the whole EBRT course. The image datasets including one simulation CT for treatment planning (initial CT) and weekly CBCT images at the time of treatment per patient were collected. For each patient, the weekly CBCT images were projected rigidly to the initial CT image using matching of the bony structures to evaluate the uterine motion during radiation treatment. To minimize internal organ motion caused by bladder, patients were asked to empty bladder first and then to drink 500 ml of water 1 h before CT simulation as well as daily radiation treatments. To maintain an empty rectum, patients were asked to have a bowel movement or to use glycerin enema within 4 h before simulation and each of the radiation treatments.

### Manual adaptive radiotherapy

**Fig. 1:**
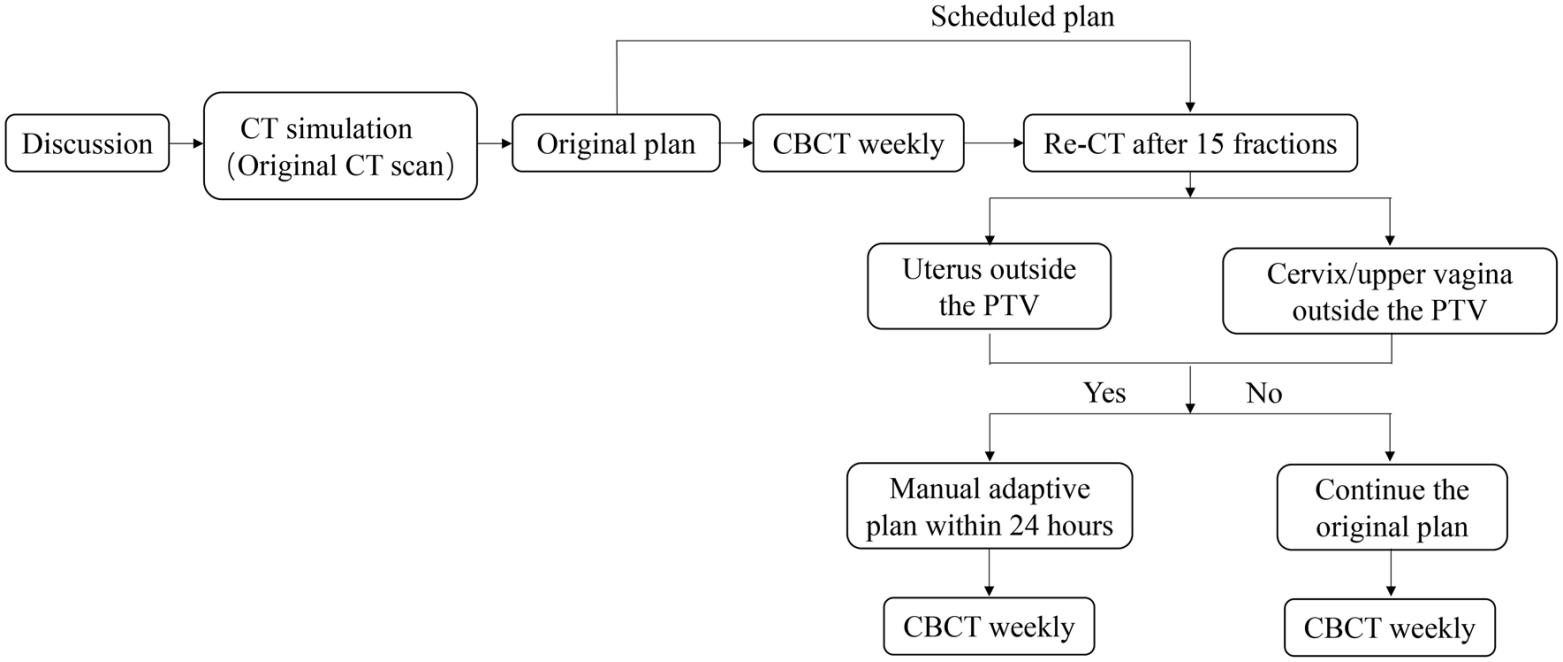
Overview of workflow procedure of the manual adaptive planning during radiotherapy

Figure 1 showed the workflow procedure of the manual adaptive planning during radiotherapy. All patients underwent a second CT simulation scan (re-CT) after 15 fractions during treatment. The re-CT images were fused to the initial CT image set using bone matching, and the original PTV were copied to the re-CT images. The thresholds for re-plan action after 15 fractions of treatment were 1) if uterus was outside the original PTV or 2) if cervix/upper vagina was outside the original PTV. In this case, a new treatment plan (the manual adaptive plan) with the re-contoured target volume and OARs based on re-CT scan of the remaining fractions was calculated. Similarly, the delineation of the clinical target volume and organs-at-risk is in accordance with RTOG consensus guidelines.

The new plan utilizes the same standards and prescribed dose as the original plan, even being created by the same physicist, the primary target coverage objectives were as follows, more than 99% of the PTV volume to be covered by 95% of the prescription dose.

### Monitoring uterine motion during external beam radiotherapy

**Fig. 2:**
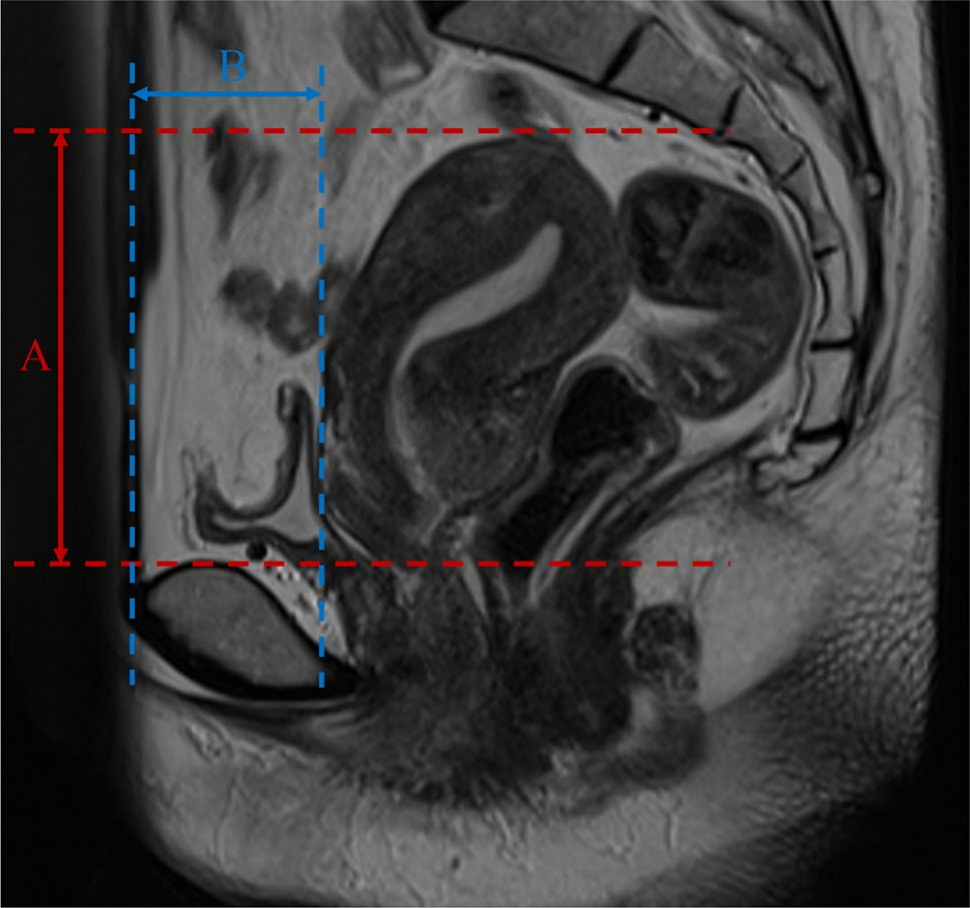
The definition of the distance to the boundary of the uterus in the median plane (sagittal plane through the midline of the body) on magnetic resonance images (an example of 1 patient, on a T2-weighted magnetic resonance image). **A** means the distance from the superior border of the fundus uteri to the superior border of the pubic symphysis. **B** means the distance from the anterior border of the uterus to the anterior border of the pubic symphysis

In the median plane (sagittal plane through the midline of the body), we measured “line A” (Fig. 2) from the superior border of the fundus uteri to the superior border of the pubic symphysis, and measured “line B” (Fig. 2) from the anterior border of the uterus to the anterior border of the pubic symphysis in weekly CBCT images as well as the initial CT image per patient. The initial position of uterus was normalized to 0. We calculated ΔA and ΔB to evaluate the movement of uterus, and eventually got value “C” for comparison.

The ΔA, ΔB and C was calculated as follows,

$$\Delta {\text{A}}\, = \,{\text{An}} - {\text{A}}0$$

$$\Delta {\text{B}}\, = \,{\text{Bn}} - {\text{B}}0$$

$${\text{C}}\, = \,\sqrt {\left( {\Delta {\text{A}}^{2} + \Delta {\text{B}}^{2} } \right)}$$

“A0” means the distance from the superior border of the fundus uteri to the superior border of the pubic symphysis in initial CT image.

“B0” means the distance from the anterior border of the uterus to the anterior border of the pubic symphysis in initial CT image.

“n” represents the number of CBCT sessions.

The volumes of cervix gross tumor were computed by eclipse software.

### Dose data acquisition

The original CT and the re-CT (15 fractions after) are fused and registered in the Varian Eclipse planning system according to the bone structure, and then the original plan is loaded onto the re-CT to generate the plan, named scheduled plan. To compare the planning target volume coverage between the manual adaptive plan and scheduled plan, the conformity index (CI) and homogeneity index (HI) were calculated. The values of dosimetric objectives obtained from the dose-volume histograms (DVHs) were compared. For the PTV, the dosimetric parameters included the mean dose (Dmean), the dose received by 98% of the target volume (D98), the dose received by 2% of the target volume (D2), homogeneity index (HI) and conformity index (CI). For the OARs, such as bowel, bladder, sigmoid colon and rectum, the dosimetric parameters referred to the maximum dose to 2 cc’s (D2cc). All these dosimetric parameters were compared between the manual adaptive plan and scheduled plan in the remaining radiation fractions after 3 weeks of treatment.

The HI was calculated as follows (Wu et al. 2003),

$${\text{HI}}\, = \,\left( {{\text{D2}} - {\text{D98}}} \right)/{\text{ Dmean}}$$

where D2, D98 and Dmean were defined as mentioned above.

The CI was calculated as follows (Paddick. 2000),

$${\text{CI}}\, = \,\left( {{\text{Vt}},{\text{ref}}/{\text{Vt}}} \right) \times ({\text{Vt}},{\text{ref}}/{\text{Vref}})$$

Vt, ref is the target volume covered by the reference isodose line, Vt is the volume of the PTV, and Vref is the total volume covered by the reference isodose line.

### Statistical analysis

The SPSS software (version 19, SPSS Inc., Chicago, IL, USA) was conducted for the statistical analysis. Kruskal–Wallis with Dunn’s multiple comparisons test were used to analyze uterine motion during EBRT, and t-tests were used to compare the accumulated doses to the targets and OARs from the two planning scenarios. In addition, the chi-square test (or Fisher’s exact test when appropriate), univariate and multivariate logistic regression analysis were performed to evaluate the clinical factors influencing target motion. We defined p < 0.05 as statistically significant. This study was approved by the Medical Ethics Committee of the Ruijin Hospital (approval number:2024153). Written informed consent was not required because of the retrospective nature of the study.

## Result

### Baseline characteristics of patients

Table S1 showed the clinical characteristics of these 82 LACC patients. The median age was 57 years (range 34–85 years) at time of diagnosis. And disease was restaged according to the 2018 FIGO staging system. The mean clinical tumor volume was 66.8cm3 (range 12–279.9cm^3^). All patients underwent a second CT simulation scan (re-CT) after 15 fractions during treatment, and 41 patients completed the remaining fractions using a new treatment plan (the manual adaptive plan) due to significant deviation of the PTV. All patients completed EBRT followed by brachytherapy, while 66 patients received concurrent platinum-based chemotherapy.

### Analysis of inter-fraction motion during EBRT

**Fig. 3:**
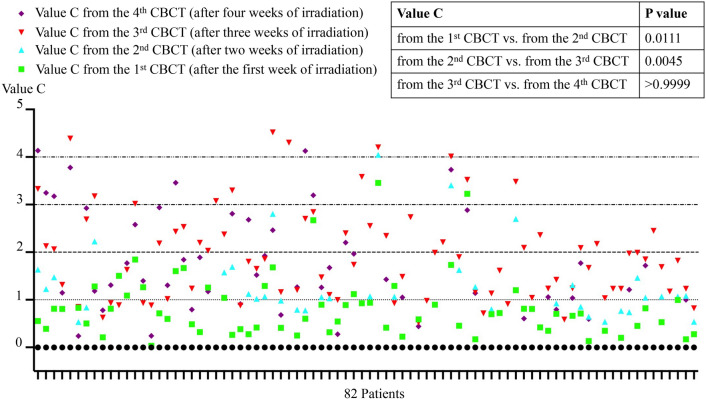
The inter-fraction motion of uterus (Value C) of all the patients. The difference of uterus positions during radiotherapy were compared by Kruskal–Wallis with Dunn’s multiple comparisons test

Table S2 summarized the motion of the uterus during the EBRT treatment period. The initial position of uterus was normalized to 0. As shown in the Table S2, the maximum movement of the uterus happened three weeks after treatment started, the median value “C” and the maximum value “C”were 1.811 and 4.517, respectively. Kruskal–Wallis with Dunn’s multiple comparisons test showed that the median uterus movement after three weeks (3rd CBCT) was greater compared with the movement after one week (1st CBCT, P < 0.0001) and two weeks (2nd CBCT, P = 0.0045) after the treatment started. Additionally, the median uterus movement after three weeks and four weeks has no significant differences. Figure 3 showed the inter-fraction motion of uterus (Value C) of all the patients.

### Target volume and OARs dose data analyses

**Fig. 4:**
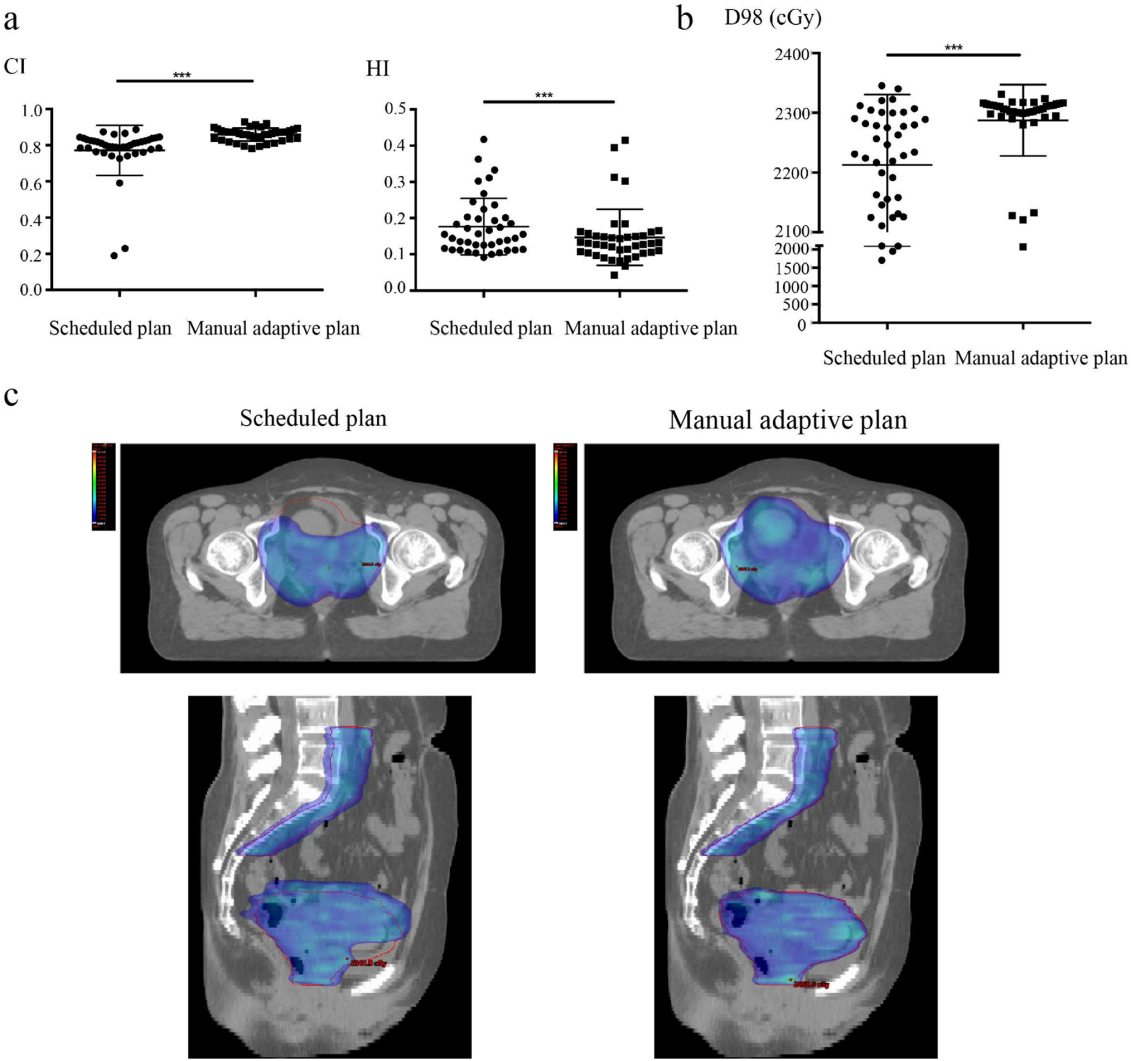
Comparison of the planning target volume coverage (PTV CI, HI, and D98) between the scheduled plan and manual adaptive plan. **a** Point plots of CI and HI for scheduled plan and manual adaptive plan, student’s t test, n = 41. **b** Point plots of accumulated dose to 98% volume for scheduled plan and manual adaptive plan, student’s t test, n = 41. **c** Comparison of the isodose distribution images of transverse for scheduled plan and manual adaptive plan of one patient. ***p < 0.001

Figure 4a showed the mean CI of scheduled plan was 0.7711 ± 0.1382, which was lower than that of the manual adaptive plan 0.8586 ± 0.0354 (P = 0.0002). Similarly, the mean HI of the scheduled plan was 0.1764 ± 0.0779, which was higher than that of the manual adaptive plan 0.1469 ± 0.0776 (P = 0.003). Additionally, as shown in the Fig. 4b, PTV D98 of manual adaptive plan was 2287.6 ± 59.8 cGy, which was higher than that of the scheduled plan (2212.9 ± 117.67 cGy, P = 0.0002). That is to say, the dose conformity, dose homogeneity and target coverage of PTV in manual adaptive plan is better than the scheduled plan (see Fig. 4c). There is no difference in D2 and Dmean between the two groups (Table S3).

Table 1 depict the differences in the D2cc of the organs-at-risk between the manual adaptive plan and scheduled plan. The D2cc of the rectum, bladder for the remaining fractions of manual adaptive plan was 2426.53 ± 67.66 cGy and 2502.75 ± 13.18 cGy, which was lower than that of scheduled plan, 2462.37 ± 74.37 cGy (P = 0.000034) and 2526.7 ± 14.32 cGy (P = 0.03), respectively. There is no difference in D2cc to the sigmoid and bowel between the two groups. The DVH lines for the two plans were displayed in Fig. S1 (an example of 1 patient).

**Table 1:** Dosimetric comparison of organs at risk between the scheduled plan and manual adaptive plan (mean ± SD)

OAR	D2cc Mean ± SD (cGy)		t value	P value
	Scheduled plan	Manual adaptive plan		
Bladder	2526.7 ± 14.32	2502.75 ± 13.18	2.247	**0.03**
Rectum	2462.37 ± 74.37	2426.53 ± 67.66	4.666	**0.000034**
Bowel	2591.65 ± 122.39	2616.54 ± 136.4	1.832	0.0743
Sigmoid	2517.51 ± 109.31	2512.79 ± 123.81	0.456	0.6506

Boldness indicates P value less than 0.05

### Relationship between clinicopathological characteristics and replanning in LACC patients

Table 2 showed the clinicopathological characteristics of patients treated with scheduled plan or manual adaptive plan. Chi-square test showed that compared with the scheduled plan group, patients in the manual adaptive plan group were younger (age ≤ 60, 73.2% vs. 48.8%, *P* = 0.024) and with larger primary tumor volume (≥ 50cm^3^, 82.9% vs. 58.5%, *P* = 0.015). In addition, parametrial infiltration was found more in manual adaptive plan than in scheduled plan one, 75.6% (31/82) vs. 51.2% (21/82), *P* = 0.022.

**Table 2:** Association between the clinicopathological characteristics and manual adaptive plan throughout radiotherapy treatment

Characteristic	Schedualed plan	Manual adaptive plan	χ^2^	P value
Age at diagnosis (year)			5.125	**0.024**
≤ 60	20 (48.8%)	30 (73.2%)		
> 60	21 (51.2%)	11 (26.8%)		
BMI			3.216	0.073
< 24	21 (51.2%)	13 (31.7%)		
≥ 24	20 (48.8%)	28 (68.3%)		
Histology			0	1
Squamous cell carcinoma	40 (97.6%)	40 (97.6%)		
Adenocarcinoma/adenosquamous carcinoma	1 (2.4%)	1 (2.4%)		
Tumor volume (cm^3^)			5.891	**0.015**
< 50	17 (41.5%)	7 (17.1%)		
≥ 50	24 (58.5%)	34 (82.9%)		
Parametrial infiltration			5.526	**0.022**
Yes	21 (51.2%)	31 (75.6%)		
No	20 (48.8%)	10 (24.4%)		
Vaginal invasion			3.418	0.332
No	4 (9.7%)	2 (4.8%)		
Upper 1/3	28 (68.3%)	27 (65.9%)		
Middle 1/3	1 (2.4%)	5 (12.2%)		
Lower 1/3	8 (19.5%)	7 (17.1%)		
LN metastases			3.124	0.077
No	24 (58.5%)	16 (39%)		
Yes	17 (41.5%)	25 (61%)		
Uterine body invasion			0.195	0.659
No	21 (51.2%)	19 (46.3%)		
Yes	20 (48.8%)	22 (53.7%)		

Boldness indicates P value less than 0.05

In univariate analysis, the age (HR 2.864; 95%CI 1.138–7.209; *P* = 0.026), primary tumor volume (HR 3.440; 95%CI 1.236–9.576; *P* = 0.018), and parametrial infiltration (HR 2.952; 95%CI 1.154–7.556; P = 0.024) were the clinical factors significantly influencing target motion during EBRT. The multivariate analysis found that the tumor volume ≥ 50cm3 (HR 3.254; 95%CI 1.062–9.969; *P* = 0.039) and parametrial infiltration (HR 3.376; 95%CI 1.229–9.270; *P* = 0.018) were independent risk factors for replanning in patients with LACC (Table 3).

**Table 3:** Univariate and multivariate logistic regression analysis of the independent risk factors for manual adaptive plan in patients with locally advanced cervical cancer (LACC)

Factor	Univariate analysis		Multivariate analysis	
	HI (95% CI)	*P*	HI (95% CI)	*P*
Age	2.864 (1.138–7.209)	0.026	2.166 (0.792–5.920)	0.132
BMI	2.262 (0.921–5.555)	0.075		
Histology	1.000 (0.060–16.548)	1.000		
Tumor volume (cm^3^)	3.440 (1.236–9.576)	0.018	3.254 (1.062–9.969)	**0.039**
Parametrial infiltration	2.952 (1.154–7.556)	0.024	3.376 (1.229–9.270)	**0.018**
Vaginal invasion	1.141 (0.689–1.888)	0.609		
LN metastases	2.206 (0.912–5.334)	0.079		
Uterine body invasion	1.216 (0.511–2.893)	0.659		

Boldness indicates P value less than 0.05

## Discussion

Substantial uterus motion and tumor shrinkage during radiotherapy significantly influence target coverage and OAR doses in LACC patients (Oh et al. 2014). There are previous studies demonstrating that oART can improve the target coverage and also reduce the dose of RT delivered to organs at risk in a variety of tumor sites (Hall et al. 2022). Online Adaptive Radiation Therapy (oART) is a radiation therapy technique that adjusts the treatment plan during the radiation therapy process based on changes in the patient's anatomical structure. This technology relies on advanced image-guided systems, such as cone-beam computed tomography (CBCT), to capture real-time images of the patient before each treatment fraction. If significant changes in the patient's anatomical structure are detected, such as tumor shrinkage, displacement, or shape alteration, the doctors can adjust the patients’ target volume and treatment plan by oART technology to ensure the accuracy and effectiveness of the treatment. During the online ART treatment, additional consideration regarding staffing and workflow are needed during each treatment, for example, it requires a radiation oncologist, medical physicist and radiation therapists to spend additional time at the machine daily (Shelley et al. 2021; Hall et al. 2022).

Thus, in our study, we introduced an alternative offline ART, the manual adaptive radiotherapy, which only required one more CT simulation during the definitive EBRT for cervical cancer. Our study analyzed the uterus mobility based on the weekly CBCT images, and found that the greatest variation in uterus position happened after 15 fractions of treatment. Thus, we recommend the patients underwent re-CT after 15 fractions during the manual adaptive radiotherapy, and ran the manual adaptive plan for the remaining fractions if significant deviation of the original PTV (1. if uterus was outside the PTV or 2. if cervix/upper vagina was outside the PTV). The new plan utilizes the same standards and prescribed dose as the original plan, even being created by the same physicist. We furthermore evaluated the potential dosimetric benefits compared with the scheduled plans, and explored the clinicopathological factors guiding patient selection for oART as well (for those have great variation in their uterus were suggested to underwent oART which will create a new plan according to their anatomical changes daily).

The key of offline ART is to determine the appropriate time for the replanning. A number of studies showed that the location of the uterus changes significantly in LACC patients during the EBRT course (Bunt et al.^2008^, Taylor et al. 2008, Jadon et al.^2014^), with the largest motion happened in the 2nd or 3rd week of treatment (Lee et al. 2007). In addition, it is reported that most of the tumor regression occurs during the first 3–4 weeks of treatment, with a mean tumor volume reduction of 50% after about 21 days, or after 30.8 Gy (Lee et al. 2004, and Bunt et al. 2006). Consistent with these findings, our study analyzed the uterus mobility based on the weekly CBCT images, and found that the largest range of uterus movement happened after 15 fractions of treatment. All patients in our study underwent re-CT simulation after 15 fractions, and half of them completed the remaining fractions using a new treatment plan (the manual adaptive plan), due to the significant changes in target volume. Several studies have analyzed the dosimetric results on target coverage and dose to OARs when various adaptive procedures adopted. Creating an adaptive plan based on weekly CT/MRI images improved the dosimetric parameters by increasing the target coverage and decreasing the dose to OARs, such as the D2cc and V45Gy (Yock et al.^2021^; Stewart et al. 2010). And the dosimetric parameters of adaptive plans based on re-CT simulation images after 15 fractions in our study were not inferior to those based on weekly CT/MRI images. We also found PTV D98 of the manual adaptive plans increased by 3.3% (2287.6 cGy vs. 2212.9 cGy, *P* = 0.0002), which was higher than that of 2.5% in the study with automated weekly replanning (Stewart et al.^2010^).

For the OARs, Yock et al. described the decrease in the D2cc of the OAR resulting from the adaptive replan for cervical cancer patients. In their study, the D2cc to the bladder, bowel, rectum, and sigmoid colon for each fraction changed an average of − 0.02 Gy (SD = 0.09, *p* = 0.017), − 0.08 Gy (SD = 0.06, *p* < 0.001), − 0.07 Gy (SD = 0.07, *p* < 0.001), and − 0.04 Gy (SD = 0.05, *p* < 0.001) respectively (Yock et al. 2021). Consistent with the previous studies, ours showed that compared to the scheduled plans, the average of D2cc to the rectum and bladder in the manual adaptive plans decreased 0.358 Gy (*P* < 0.001) and 0.240 Gy (*P* = 0.03), respectively. However, there is no difference in D2cc to the sigmoid or bowel between the two plans, indicating the manual adaptive plan, which depends on numerous interrelated considerations regarding plan reoptimization, may not universally bring dosimetric benefits for all organs in all situations. On the contrary, Oh et al. analyzed the dosimetric effects of various adaptation strategies for cervix cancer. They observed dose reduction to the OAR was not at the level of clinically meaningful in most cases, though target shrinkage was observed (Oh et al. 2014). Therefore, the dosimetric advantages of manual adaptive plans in patients with LACC needed further validation in large-scale studies.

Unfortunately, financial and human resources remain barriers for the introduction of online adaptive radiotherapy in most low- and middle-income countries where cervical cancer has a greater prevalence, although the online ART improved CTV D98 and reduced normal tissue dose. In our study, we meant to find out who would benefit the most from the adaptive radiotherapy with daily replanning to save the time as well as human resources spending on the ART for all the patients. Thus, it’s necessary to identify patients who experience greater organ mobility during EBRT. In our study, the manual adaptive replanning workflow started when the uterus position observed beyond the PTV margin by re-CT after 15 fractions. We tried to explore the clinicopathological factors that may influence organ mobility during EBRT to establish a prediction model selecting patients the most suitable for the ART, which has not been reported in previous studies. The patients in our study with bulky mass (≥ 50cm^3^, OR = 3.254, *P* = 0.039) or with parametrial infiltration (OR = 3.376, *P* = 0.018) were highly suggested to receive adaptive replanning during definitive radiotherapy. Data from our study revealed that 82.9% of patients with tumor volume ≥ 50 cm^3^ and 75.6% of patients with parametrial infiltration received raplanning. However, to date, there has been no study on the relationship between clinicopathological factors and organ mobility during EBRT. Therefore, further studies are needed to verify the value of these factors for predicting ART in patients with LACC.

The limitations of this study are the CBCT-guided ART and the limited sample size. However, kV CBCT is currently the most commonly available for online volumetric imaging, and in most cases differentiating target from surrounding tissues was not difficult (Khan et al. 2012). The MRI-guided online ART system has the potential advantages such as excellent soft-tissue definition and more accurate target delineation compared to CBCT-guided ART (Fields et al. 2016). And the results from our study need to be confirmed in a large sample of prospective studies. Despite these limitations, our study determined the appropriate time of manual adaptive replanning and further revealed that the tumor volume and parametrial infiltration may be two factors that predict the need for online ART during the EBRT for LACC patients, which has great significance.

In summary, we found the most significant organ motion happened after 15 fractions during treatment. The manual adaptive plan ran only once during the whole treatment time improved the dose coverage and decreased the volume of organs at risk in patients with LACC. Patients with bulky mass or with parametrial infiltration were highly suggested to receiving adaptive radiotherapy.

### Supplementary Information

Below is the link to the electronic supplementary material.

Supplementary file1 (JPG 184 KB)

Supplementary file2 (DOCX 15 KB)

Supplementary file3 (DOCX 20 KB)

## Data Availability

The data that support the findings of this study are available from the corresponding author, upon reasonable request.
