# Supplementary figures and images for: The clinical practice and dosimetric outcome of the manual adaptive planning during definitive radiotherapy for cervical cancer
Source: J Cancer Res Clin Oncol. 2024 May 27;150(5):280. doi: 10.1007/s00432-024-05809-z (PMC11130034; doi:10.1007/s00432-024-05809-z)

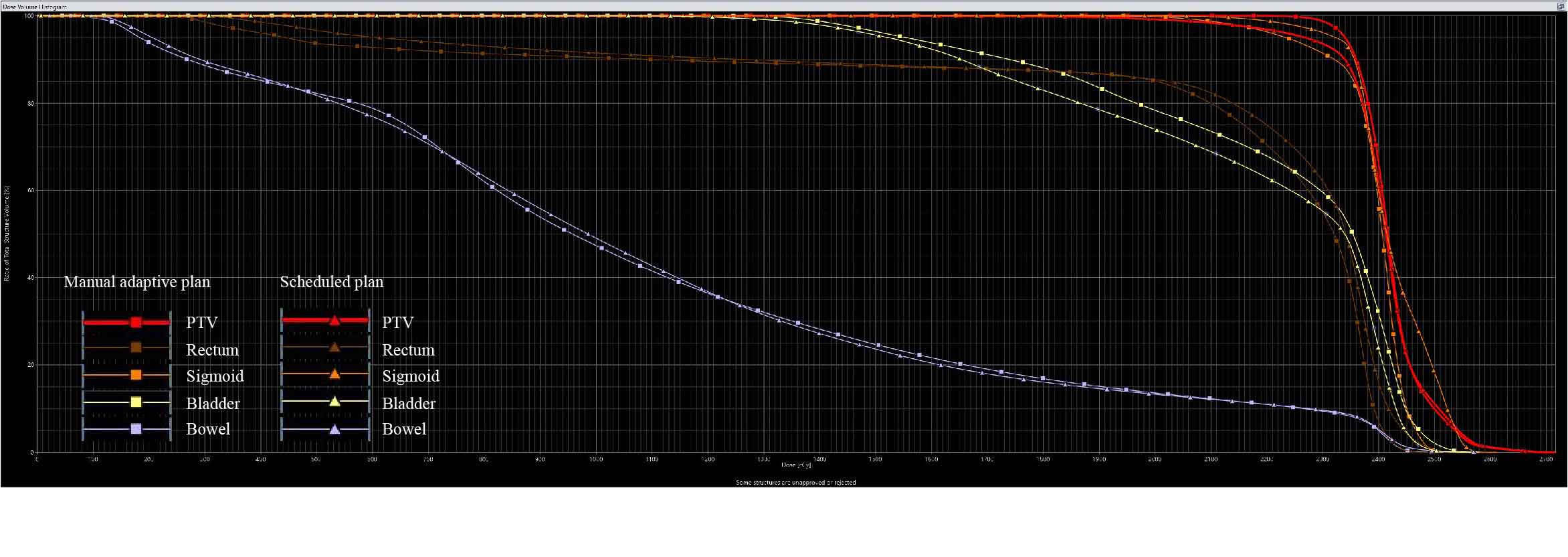

Supplement: Supplementary file 1 — Supplementary file1 (JPG 184 KB) [file 432_2024_5809_MOESM1_ESM.jpg]
