# Supplementary material for: The clinical practice and dosimetric outcome of the manual adaptive planning during definitive radiotherapy for cervical cancer
Source: J Cancer Res Clin Oncol. 2024 May 27;150(5):280. doi: 10.1007/s00432-024-05809-z (PMC11130034; doi:10.1007/s00432-024-05809-z)
Supplement: Supplementary file 2 — Supplementary file2 (DOCX 15 KB) [file 432_2024_5809_MOESM2_ESM.docx]

**Supplementary material**

Figure S1. Comparison of the dose volume histograms (DVHs) for the manual adaptive plan and the scheduled plan of one patient (). In the patients, D98 of scheduled plan was 2131.0cGy, which was lower than that of manual adaptive plan 2313.6cGy, D95 of scheduled plan was 2268.6cGy, which was lower than that of manual adaptive plan 2340cGy, indicating that the target coverage of PTV in scheduled plan was worse than that of the manual adaptive plan. Additionally, the dvh line of rectum and sigmoid for manual adaptive plan was lower than that of scheduled plan, but the dvh line of bladder for the scheduled plan was lower, and the dvh line of bowel of these two plans had no significant deviation.
