# Supplementary material for: The clinical practice and dosimetric outcome of the manual adaptive planning during definitive radiotherapy for cervical cancer
Source: J Cancer Res Clin Oncol. 2024 May 27;150(5):280. doi: 10.1007/s00432-024-05809-z (PMC11130034; doi:10.1007/s00432-024-05809-z)
Supplement: Supplementary file 3 — Supplementary file3 (DOCX 20 KB) [file 432_2024_5809_MOESM3_ESM.docx]

Table S1. Patients and treatment characteristics.

| Characteristic | Characteristic | Patients (n=82) |
| --- | --- | --- |
| Age at diagnosis (year) | ≤60 | 50 |
|  | >60 | 32 |
|  | Median (Range) | 57 (34-85) |
| BMI | ＜24 | 34 |
|  | ≥24 | 48 |
| Histology | Squamous cell carcinoma | 80 |
|  | Adenocarcinoma/adenosquamous carcinoma | 2 |
| FIGO stage | IB-IIA | 12 |
|  | IIB | 9 |
|  | IIIA | 8 |
|  | IIIB | 7 |
|  | IIIC | 37 |
|  | IVA | 9 |
| Tumor volume (cm^3^) | ＜50 | 24 |
|  | ≥50 | 58 |
|  | Median (Range) | 66.8 (12-279.9) |
| Parametrial infiltration | Yes | 52 |
|  | No | 30 |
| LN metastases | Yes | 42 |
|  | No | 40 |
| EBRT dose（Gy） | Median | 50.4 |
|  | Range | 45-50.4 |
| Uterine body invasion | Yes | 42 |
|  | No | 40 |
| Vaginal invasion | No | 6 |
|  | Upper 1/3 | 55 |
|  | Middle 1/3 | 6 |
|  | Lower 1/3 | 15 |
| Concurrent chemotherapy | YES | 66 |
|  | NO | 16 |

Table S2. Measurement of the difference of uterus positions during radiotherapy

|  | 1st CBCT | 2nd CBCT | 3rd CBCT | 4th CBCT |
| --- | --- | --- | --- | --- |
| ΔA |  |  |  |  |
| Median | -0.2050 | -0.400 | -0.840 | -0.9050 |
| Range | -3.440-2.950 | -3.790-0.750 | -4.360-1.770 | -4.050-1.010 |
| ΔB |  |  |  |  |
| Median | -0.1000 | 0.0900 | 0.5000 | 0.7500 |
| Range | -1.230-2.390 | -2.130-2.350 | -3.090-3.330 | -1.180-3.950 |
| C |  |  |  |  |
| Median | 0.7080 | 1.066 | 1.811 | 1.429 |
| Range | 0.03162-3.457 | 0.5334-4.047 | 0.5197-4.517 | 0.2377-4.134 |

Table S3. Comparison of the planning target volume coverage (PTV CI, HI, and D98) between the scheduled plan and manual adaptive plan

|  | Scheduled plan  Mean±SD | Manual adaptive plan  Mean±SD | t value | P value |
| --- | --- | --- | --- | --- |
| PTV CI | 0.7711±0.1382 | 0.8586±0.0354 | 4.0282 | 0.0002 |
| PTV HI | 0.1764±0.0779 | 0.1469±0.0776 | 3.1567 | 0.003 |
| PTV D98 | 2212.9±117.67 | 2287.6±59.8 | 4.0515 | 0.0002 |
| PTV D2 | 2640.79±109.13 | 2644.6±131.2 | 0.3584 | 0.7219 |
| PTV Dmean | 2431.2±44.82 | 2436.47±43.0 | 1.1193 | 0.2697 |
